# Pupillometry changes following ocular administration of brimonidine in patients experiencing acute migraine attack

**DOI:** 10.1097/MD.0000000000043292

**Published:** 2025-07-11

**Authors:** Caner Karakaya, Seyma Kilic, Isik Yildirim

**Affiliations:** a Department of Ophthalmology, Istanbul Medipol University, Faculty of Medicine, Istanbul, Turkey; b Department of Neurology, Istanbul Medipol University, Faculty of Medicine, Istanbul, Turkey.

**Keywords:** brimonidine, infrared pupillometry, migraine ocular findings, migraine pain, VAS scores

## Abstract

**Background:**

This study aimed to assess pupillometry changes and alterations in clinical headache severity following brimonidine tartrate 0.15% administration in patients with acute migraine attacks.

**Methods:**

A randomized controlled prospective study was conducted involving 42 patients with acute migraine attacks and 48 healthy individuals in the control group. Infrared pupillometry and Visual Analog Scale measurements were assessed before and after ocular instillation of brimonidine tartrate 0.15%.

**Results:**

In patients with acute migraine attacks, the mean latency was 156 ms compared with 173 ms in the control group (*P* = .042). Pupillary amplitude was 2.45 mm in the acute migraine group and 2.91 mm in the control group (*P* = .041). Both groups exhibited a statistically significant decrease in pupillary diameter after the administration of brimonidine tartrate 0.15% in dynamic and static pupillometry. However, latency was significantly prolonged in the acute migraine group (*P* = .009), whereas no significant change was observed in the control group (*P* > .05). Although there was a statistically significant decrease in the anterior chamber depth in the migraine group after brimonidine tartrate 0.15% (*P* = .005), the decrease in the control group was not significant (*P* = .052). Visual Analog Scale scores significantly decreased in patients with acute migraine patients after brimonidine (*P* < .001).

**Conclusion:**

The study findings suggest that patients experiencing acute migraine attacks demonstrate shorter pupillary constriction latencies, indicating the presence of accompanying sympathetic hypoactivity in migraine patients. Latency prolongation after brimonidine suggests sympathetic hyperfunction secondary to chronic sympathetic hypofunction in migraine patients. Brimonidine, with its ability to cross the blood–brain barrier, may provide pain relief through a mechanism similar to that of latency prolongation. This study sheds light on the potential role of brimonidine tartrate in the management of acute migraine attacks, and highlights its effects on pupillometric parameters, thereby contributing to our understanding of migraine pathophysiology and potential treatment avenues.

## 1. Introduction

The assessment of pupillometry results provides valuable insights into the function of the autonomic nervous system (ANS) under various neurological conditions. Pupillary measurements conducted using portable pupillometers have been widely used in intensive care settings to evaluate ANS function in various neurological diseases. This study employed infrared pupillometry, a non-portable, noninvasive method that delivers quantitative and objective measurements of static and dynamic parameters with minimal error.

Migraine is a chronic and lifelong condition that affecting >1 billion individuals across all geographic regions worldwide.^[1,2]^ ANS dysfunction is not the cause of migraine; however, ANS findings accompany the disease in 82% of migraine patients. These findings mainly originate from the parasympathetic system and include lacrimation, conjunctival congestion, eyelid edema, ear fullness, and nasal congestion.^[3]^

In this study, brimonidine tartrate 0.15%, an alpha-2 agonist, was used to explore the pupillometric changes in patients with migraine attacks. While previous studies have examined the effects of aproclonidine,^[4]^ there is a notable absence of studies investigating brimonidine in this context. Although both drugs are alpha-2 agonists, their pupillary and ocular effects significantly differ. Brimonidine, with its higher selectivity for alpha-2 receptors, induces miosis (pupil constriction) upon ocular instillation, whereas aproclonidine causes mydriasis (pupil dilation).^[5–7]^ Additionally, aproclonidine causes upper eyelid retraction, a phenomenon not observed with brimonidine.^[8,9]^

This study hypothesizes that significant ANS changes occur in migraine patients during attacks compared to healthy individuals and that brimonidine administration will produce distinct pupillometric findings in migraine patients relative to a control group.

## 2. Methods

This randomized controlled prospective study was conducted at a university hospital, adhering to the principles outlined in the Declaration of Helsinki. Approval was granted by the Istanbul Medipol University Ethics Committee (approval number: 1008; date: November 24, 2022). Written informed consent was obtained from all patients prior to measurement.

Participants underwent neurological and ocular examinations between January 2023 and April 2024. Individuals with any ocular disease, such as glaucoma, cataracts, optic neuropathy, or retinal disorders, as well as those with non-migraine neurological conditions, were excluded from the study. A total of 84 eyes from 42 patients with acute migraine pain and 96 eyes from 48 age-matched healthy controls were included in the study. The study was a parallel-group, randomized controlled trial with a 1:1.14 allocation ratio. The random allocation sequence was generated, participants were enrolled, and participants were assigned to interventions by the corresponding author.

Following neurological examination, participants underwent pupillometry assessments in the ophthalmology department. Infrared pupillometry were performed using the Aladdin HW 3.0 (Topcon, Tokyo, Japan) device, capable of measuring pupil sizes between 0.5 mm and 10 mm. Initial measurements were obtained after participants had adapted to darkness for at least 1 minute. Subsequently, brimonidine tartrate 0.15% (Alphagan^®^ P, Allergan Sales LLC, Waco) ophthalmic solution was instilled into the inferior fornix of both eyes. A second set of measurements was conducted 30 minutes after the instillation. Measurements from the right eye of all the patients were used as the basis for evaluating the study results.

Initially, Visual Analog Scale (VAS) scores were not assessed in patients. However, when pain reduction was observed in those administered brimonidine, the VAS scores before and after brimonidine administration were subsequently evaluated in 33 out of 42 patients experiencing migraine attacks. Pain levels were assessed by participants rating their pain on a scale of 1 to 10 before and 1 hour after receiving a single dose of brimonidine. Other medications including painkillers were not administered before or after pupillometry and VAS assessments.

Dynamic pupillometry involves rapidly altering the light intensity to measure the minimum and maximum pupil diameters in millimeters. Latency, measured in milliseconds, represents the time from light onset to pupillary constriction. Static pupillometry maintained light intensity under photopic and mesopic conditions for a set duration by measuring the mean pupillary diameter.

**Figure 1.**
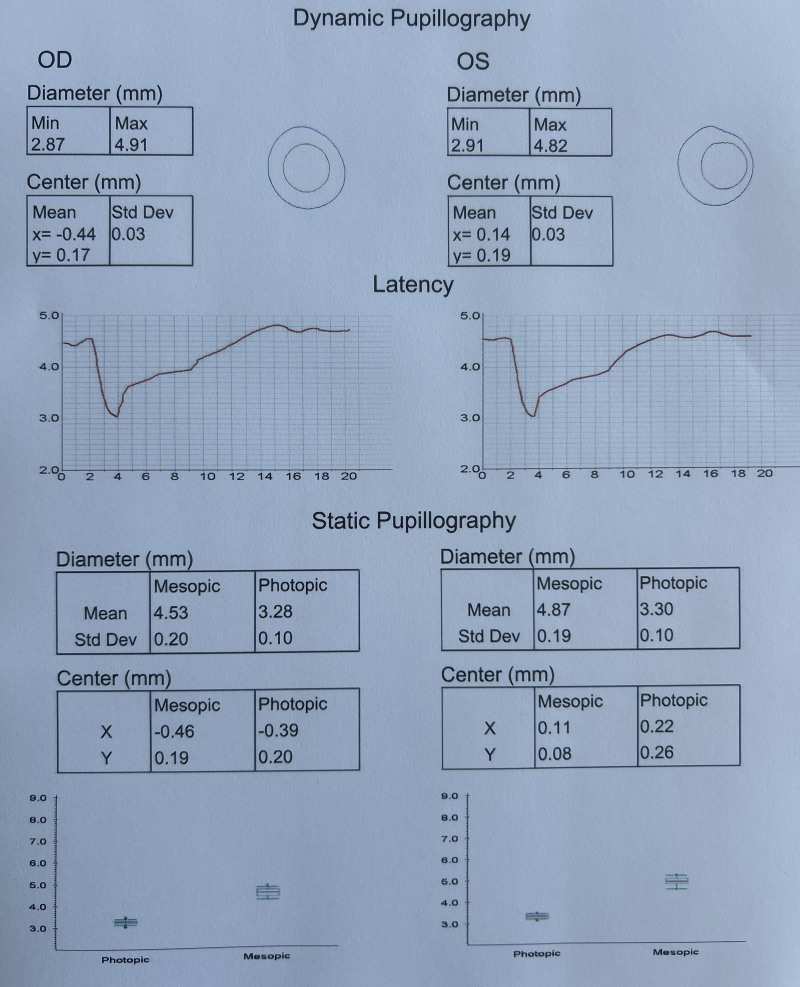
The output of the Topcon Aladdin pupillometer system displaying dynamic and static pupillometry, along with the latency graph. During dynamic pupillometry, the device rapidly changes light intensity and calculates the maximum and minimum pupil diameters in both the right and left eyes. Latency is defined as the time interval between the first light stimulus and the onset of pupil constriction. In static pupillometry, the light intensity remains constant under photopic and mesopic conditions, and the pupil diameter is measured accordingly in both states.

Figure 1 depicts the participants’ Topcon Aladdin pupillometry measurements and illustrates the dynamic and static pupillometry parameters assessed in this study

### 2.1. Statistical analyses

Statistical analysis was performed using IBM SPSS version 25, with a significance level set at *P* < .05 for two-tailed tests. Descriptive statistics were used to summarize the means, standard deviations, and percentages of sample data. Before performing statistical tests on each parameter, the normality of the data was assessed using the Shapiro–Wilk test to determine its suitability for the *t* test.

Paired samples *t* tests were used to evaluate the changes in pupillometry, anterior chamber depth, and latency before and after brimonidine tartrate administration. The independent samples *t* test was used to compare the migraine and control groups. Levene test for equality of variances was also used to assess whether the variances were equal in independent *t* tests.

The difference between VAS scores before and after brimonidine tartrate administration was assessed using a paired-samples *t* test.

## 3. Results

The study included 42 participants with acute migraine attacks, comprising 28 women and 14 men, with a mean age of 33.5 years (range 21–49 years). The control group consisted of 48 individuals (24 men and 24 women) with a mean age of 32.2 years (range 20–48 years). No statistically significant differences were observed between the 2 groups in terms of sex or age (*P* = .111 and *P* = .661, respectively).

All participants had a decimal visual acuity of 1.0 in both eyes. The spherical equivalent refractive errors of the participants’ right eyes were −0.69 diopters (range +2.50 to −3.25 diopters) in the migraine group and −0.12 diopters (range +2.75 to −3.75 diopters) in the control group. There was no significant difference in the refractive error between the 2 groups (*P* > .154).

Mean latency was 156 ms in the acute migraine group and 173 ms in the control group. A statistically significant difference in latency was detected between the 2 groups (*P* = .042) (Table 1).

**Table 1:** Pupillometry results before brimonidine instillation in groups (mean ± SD). *P*-values were calculated using independent samples *t* test.

	Acute migraine attack group	Control group	*P* value
Minimum pupillary diameter in dynamic pupillometry	2.86 ± 0.53 mm	2.38 ± 0.70 mm	*P* = .02
Maksimum pupillary diameter in dynamic pupillometry	5.31 ± 0.71 mm	5.30 ± 0.74 mm	*P* = .953
Mean mesopic pupillary diameter in static pupillometry	4.73 ± 0.77 mm	4.84 ± 0.89 mm	*P* = .678
Mean photopic pupillary diameter in static pupillometry	3.43 ± 0.37 mm	3.23 ± 0.57 mm	*P* = .205
Latance (millisecond)	156 ± 27 ms	173 ± 22 ms	*P* = .042
Amplitude of pupillary contraction	2.45 ± 0.58 mm	2.91 ± 0.79 mm	*P* = .041

Dynamic pupillometry measurements showed that the amplitude, calculated as the difference between the maximum and minimum pupillary widths (was 2.45 mm in the acute migraine group and 2.91 mm in the control group). A statistically significant difference in amplitude was observed between the groups (*P* = .041) (Table 1).

After the instillation of brimonidine, dynamic pupillometry results indicated a significant decrease in both the minimum and maximum pupillary diameters in the acute migraine group (both *P* < .001). A similar significant decrease was observed in the control group (*P* = .039 and *P* < .001) for the minimum and maximum diameters, respectively (Table 2).

**Table 2:** Dynamic pupillometry, static pupillometry, latency, anterior chamber depth, amplitude changes before and after brimonidine tartrate 0.15% (mean ± SD). Pupillometry values are average millimeter values. The terms pre and post mean before and after brimonidine tartrate instillation. *P*-values were calculated using paired samples *t* test.

	Acute migraine attack group		*P* value	Control group		*P* value
	Prebrimonidine	Postbrimonidine		Prebrimonidine	Postbrimonidine	
Dynamic pupillometry						
Minimum pupillary diameter	2.86 ± 0.53 mm	2.34 ± 0.51 mm	<.001	2.38 ± 0.70 mm	2.19 ± 0.53 mm	.039
Maximum pupillary diameter	5.31 ± 0.71 mm	4.17 ± 0.96 mm	<.001	5.30 ± 0.74 mm	4.00 ± 0.85 mm	<.001
Static pupillometry						
Mean photopic diameter	3.43 ± 0.37 mm	3.04 ± 0.53 mm	.001	3.23 ± 0.57 mm	2.84 ± 0.41 mm	.001
Mean mesopic diameter	4.73 ± 0.77 mm	3.81 ± 0.94 mm	<.001	4.84 ± 0.89 mm	3.55 ± 0.82 mm	<.001
Latency (millisecond)	156 ± 27 ms	172 ± 24 ms	.009	173 ± 22 ms	167 ± 24 ms	.407
Anterior chamber depth	3.44 ± 0.27 mm	3.42 ± 0.27 mm	.005	3.43 ± 0.24 mm	3.42 ± 0.23 mm	.052
Amplitude	2.45 ± 0.58 mm	1.82 ± 0.68 mm	<.001	2.91 ± 0.79 mm	1.81 ± 0.54 mm	<.001

In static pupillometry, a significant reduction was noted in the mean photopic and mesopic pupillary diameters in the acute migraine group following brimonidine instillation (*P* = .001 and *P* < .001, respectively). A significant decrease was also observed in the mean photopic and mesopic pupillary diameters in the control group after brimonidine instillation (*P* = .001 and *P* < .001, respectively) (Table 2).

Significant changes in latency were observed in the acute migraine group after brimonidine instillation. Latency increased by an average of 16 milliseconds (ms), with a statistically significant difference (*P* = .009). Conversely, the control group experienced an average latency decrease of 6 ms, but no significant difference was found in the latency before and after instillation (*P* = .407) (Table 2).

Both acute migraine and control groups exhibited a decrease in anterior chamber depth following brimonidine instillation. This reduction was statistically significant in the migraine group, but not in the control group. (*P* = .005 and *P* = .052, respectively) (Table 2).

VAS scores were evaluated in 33 of the 42 patients with migraine attacks; this group included 24 women and 9 men. The mean VAS score before brimonidine administration was 6.90 (range, 5–9), whereas the mean VAS score 1 hour after brimonidine was 5.21 (range, 0–8). The change in the VAS score was statistically significant (*P* < .001). Specifically, the VAS scores remained unchanged in 9 of the 32 patients, increased in 2, and decreased in 21 (Table 3).

**Table 3:** Visual Analog Scores (VAS) of 52 patients with acute migraine before and 1 hour after brimonidine tartrate 0.15% (mean ± SD). n1 indicates the number of patients whose pain decreased, n2 the number of patients whose pain did not change, and n3 the number of patients whose pain increased.

	VAS score	n1	n2	n3
Before brimonidine tartrate	6.96 ± 1.15			
After brimonidine tartrate	5.11 ± 2.03	21 (66%)	9 (28%)	2 (6%)

## 4. Discussion

Recent community meta-analyses and population-based reviews have indicated that migraine affects approximately 11.6% of the global population, impacting over 1 billion people worldwide.^[10,11]^ Despite numerous studies, the precise pathophysiology of migraines remains unclear. The ANS has been hypothesized to play a significant role in this disease, with pupillary light reflexes assessed during both the migraine attack and attack-free periods. However, the findings of various studies have been controversial.^[4,12,13]^

Pupillary size results from the interplay between sympathetic and parasympathetic systems. Specifically, pupillary dilation velocity reflects sympathetic activity, whereas latency, amplitude, and contraction velocity reflect parasympathetic activity.^[14–16]^

Kavuncu et al investigated the role of the ANS in patients with migraine and compared pupillometry results during attack-free periods with those of a control group. They found that, despite being attack-free, migraine patients had a significantly lower pupillary constriction latency during dynamic pupillometry than the control group (*P* = .001). This result was interpreted as a disruption in the sympathetic-parasympathetic balance, favoring parasympathetic activity in patients.^[17]^ In our study, pupillary constriction latency was also lower in patients with migraine during attacks (156 ms vs 173 ms in the migraine and control groups, respectively), with a significant difference between groups (*P* = .042).

Kavuncu et al also reported that, while the pupillary constriction amplitude was lower in migraine patients than in controls, this difference was not statistically significant (*P* = .211). In our study, the amplitude was 2.45 mm in the migraine group and 2.91 mm in the control group, with a significant difference observed (*P* = .041). This discrepancy may be attributable to the focus of our study on patients with acute migraine attacks, whereas Kavuncu et al assessed patients during pain-free periods. The reduced amplitude in migraine patients could reflect hypofunction of both the sympathetic and parasympathetic systems. Furthermore, in our study, the finding that the minimum pupillary diameter was 2.86 mm in the migraine group and 2.38 mm in the control group (*P* = .02) supports the theory that parasympathetic hypoactivity is also present in migraine patients.

In contrast, Cambron et al did not find differences in latency and amplitude between the ictal and interictal periods in migraine patients compared to controls.^[4]^ They observed an increase in latency only after instilling aproclonidine 1% drops during the ictal phase (*P* = .022 for both eyes). They attributed this latency increase to parasympathetic reduction following aproclonidine instillation, suggesting that parasympathetic hypofunction could result from long-standing sympathetic hypersensitivity in patients with migraine. The absence of differences in latency and amplitude in their study may be because of the use of the Procyon P3000 pupillometer, which measures both eyes simultaneously, unlike the Topcon pupillometry used in our study.

Brimonidine known to induces miosis primarily through its alpha-2 adrenergic effects.^[5,7]^ Unlike brimonidine, aproclonidine does not induce miosis.^[18]^ Although both drugs are alpha-2 adrenergic agonists, the higher selectivity of brimonidine for alpha-2 receptors and the minimal alpha-1 adrenergic effects account for their distinct impact. Higher alpha-2 selectivity prevents mydriasis and lid retraction, and its less polar molecular structure allows it to cross the blood–brain barrier more readily.^[9,18,19]^

Our study is unique in that it demonstrated pupillometric changes during acute migraine attacks using the highly selective alpha-2 agonist brimonidine. In the acute migraine group, the latency increased from 156 ms to 172 ms after brimonidine administration (*P* = .009). In contrast, the latency of the control group decreased from 173 ms to 167 ms (*P* = .407). This finding suggests that brimonidine increases parasympathetic activity and decreases latency in healthy individuals, whereas in patients with acute migraine, prolongation of latency may be due to sympathetic hypersensitivity resulting from long-term sympathetic hypofunction. An increase in latency following brimonidine drops may aid in the differential diagnosis of migraine in patients with headache. Further studies should investigate pupillometry findings after brimonidine tartrate administration in other common headache types such as cluster headache and hypertension-related headache.

Another significant finding of our study is the notable reduction in pain in migraine patients after administration of brimonidine, a commonly used glaucoma medication. Although the pain reduction effect was not anticipated at the outset of the study, VAS scores were subsequently evaluated in patients following brimonidine drops without the use of any painkillers. VAS scores were assessed in 33 of the 42 patients with migraine, showing a mean score reduction from 6.90 (range: 5–9) before brimonidine to 5.21 (range: 0–8) 1 hour after administration. This change was statistically significant (*P* < .001). This pain relief may be due to the ability of brimonidine to enter the circulation through episcleral veins and cross the blood–brain barrier, reducing pain through its less polar structure. The mechanism of pain reduction could be related to post-drop sympathetic hypersensitivity resulting from chronic sympathetic hypofunction in migraine patients. Brimonidine may represent a viable and easy-to-use alternative for treating acute migraine attacks. However, further placebo-controlled studies are required to confirm its efficacy in reducing pain in acute migrain patients.

## 5. Conclusion

Objectively different results were observed in pupillometry in patients with acute migraine attacks compared with the control group. In addition, both latency prolongation and pain reduction were observed in the patients treated with brimonidine.

Moreover, the shorter pupillary constriction latency observed in patients with acute migraine is consistent with the association of ANS findings in migraines. The prolongation of latency following brimonidine administration suggests sympathetic hyperfunction, potentially resulting from chronic sympathetic hypofunction in migraine patients.

## Author contributions

**Conceptualization:** Caner Karakaya.

**Data curation:** Caner Karakaya, Seyma Kilic, Isik Yildirim.

**Formal analysis:** Caner Karakaya, Isik Yildirim.

**Investigation:** Caner Karakaya, Seyma Kilic, Isik Yildirim.

**Methodology:** Caner Karakaya, Isik Yildirim.

**Project administration:** Caner Karakaya.

**Software:** Caner Karakaya, Seyma Kilic.

**Supervision:** Caner Karakaya, Isik Yildirim.

**Validation:** Caner Karakaya, Seyma Kilic.

**Visualization:** Caner Karakaya, Seyma Kilic.

**Writing – original draft:** Caner Karakaya, Seyma Kilic.

**Writing – review & editing:** Caner Karakaya, Isik Yildirim.

## References

[1] Vos T Abajobir AA Abate KH. Global, regional, and national incidence, prevalence, and years lived with disability for 328 diseases and injuries for 195 countries, 1990–2016: a systematic analysis for the Global Burden of Disease Study 2016. Lancet. 2017;390:1211–59. 28919117 10.1016/S0140-6736(17)32154-2 PMC5605509

[2] Ashina M. Migraine. N Engl J Med. 2020;383:1866–76. 33211930 10.1056/NEJMra1915327

[3] Straube A Ellrich J Eren O Blum B Ruscheweyh R. Treatment of chronic migraine with transcutaneous stimulation of the auricular branch of the vagal nerve. J Headache Pain. 2015;16:543. 26156114 10.1186/s10194-015-0543-3 PMC4496420

[4] Cambron M Maertens H Paemeleire K Crevits L. Autonomic function in migraine patients:ictal and interictal pupillometry. Headache. 2014;54:655–62. 23808550 10.1111/head.12139

[5] McDonald JE 2nd El-Moatassem Kotb AM Decker BB. Effect of brimonidine tartrate ophthalmic solution 0.2% on pupil size in normal eyes under different luminance conditions. J Cataract Refract Surg. 2001;27:560–564. 11311624 10.1016/s0886-3350(01)00769-6

[6] Mastropasqua L Carpineto P Ciancaglini M. Brimonidine and pupillary diameter. Ophthalmology. 1998;105:1352–3. 9709738 10.1016/S0161-6420(98)98006-X

[7] Kesler A Shemesh G Rothkoff L Lazar M. Effect of brimonidine tartrate 0.2% ophthalmic solution on pupil size. J Cataract Refract Surg. 2004;30:1707–10. 15313294 10.1016/j.jcrs.2004.02.043

[8] Yuksel N Guler C Caglar Y Elibol O. Apraclonidine and clonidine: a comparison of efficacy and side effects in normal and ocular hypertensive volunteers. Int Ophthalmol. 1992;16:337–42. 1358852 10.1007/BF00917987

[9] Tuncer I Bilgin S Zengin MO. Effect of brimonidine tartrate 0.15% on scotopic pupil size and upper eyelid position: controlled trial. Eye (Lond). 2021;35:672–5. 32518394 10.1038/s41433-020-1007-9 PMC8027394

[10] Woldeamanuel YW Cowan RP. Migraine affects 1 in 10 people worldwide featuring recent rise: a systematic review and meta-analysis of community-based studies involving 6 million participants. J Neurol Sci. 2017;372:307–15. 28017235 10.1016/j.jns.2016.11.071

[11] Ashina M Katsarava Z Do TP. Migraine: epidemiology and systems of care. Lancet. 2021;397:1485–95. 33773613 10.1016/S0140-6736(20)32160-7

[12] Gotoh F Komatsumoto S Araki N Gomi S. Noradrenergic nervous activity in migraine. Arch Neurol. 1984;41:951–5. 6477230 10.1001/archneur.1984.04050200057018

[13] Mylius V Braune HJ Schepelmann K. Dysfunction of the pupillary light reflex following migraine headache. Clin Auton Res. 2003;13:16–21. 12664243 10.1007/s10286-003-0065-y

[14] Tekin K Sekeroglu MA Kiziltoprak H Doguizi S Inanc M Yilmazbas P. Static and dynamic pupillometry data of healthy individuals. Clin Exp Optom. 2018;101:659–65. 29356077 10.1111/cxo.12659

[15] Micieli G Tassorelli C Martignoni E Marcheselli S Rossi F Nappi G. Further characterization of autonomic involvement in multiple system atrophy:a pupillometric study. Funct Neurol. 1995;10:273–80. 8837991

[16] Smith SA Smith SE. Bilateral Horner’s syndrome:detection and occurrence. J Neurol Neurosurg Psychiatry. 1999;66:48–51. 9886450 10.1136/jnnp.66.1.48 PMC1736154

[17] Kavuncu SK Nalcacioglu P Gunes NH Ozkoyuncu D Kara C. A shift of the pupillary balance towards the parasymphathetic system in migraine patients with aura. Noro Psikiyatr Ars. 2022;59:268–73. 36514515 10.29399/npa.27856 PMC9723838

[18] Meyler’s Side Effects of Drugs: The International Encyclopedia of Adverse Drug Reactions and Interactions. 16th ed. 2016:1050.

[19] Scheinfeld N. The use of apraclonidine eyedrops to treat ptosis after the administration of botulinum toxin to the upper face. Dermatol Online J. 2005;11:9. 15748550

